# Soft Robots with Plant‐Inspired Gravitropism Based on Fluidic Liquid Metal

**DOI:** 10.1002/advs.202306129

**Published:** 2024-03-06

**Authors:** Gangsheng Chen, Biao Ma, Yi Chen, Yanjie Chen, Jin Zhang, Hong Liu

**Affiliations:** ^1^ State Key Laboratory of Digital Medical Engineering School of Biological Science and Medical Engineering Southeast University Nanjing 210096 China

**Keywords:** bioinspired soft robot, electronics‐free robots, gravitropism, liquid crystal elastomer, liquid metal

## Abstract

Plants can autonomously adjust their growth direction based on the gravitropic response to maximize energy acquisition, despite lacking nerves and muscles. Endowing soft robots with gravitropism may facilitate the development of self‐regulating systems free of electronics, but remains elusive. Herein, acceleration‐regulated soft actuators are described that can respond to the gravitational field by leveraging the unique fluidity of liquid metal in its self‐limiting oxide skin. The soft actuator is obtained by magnetic printing of the fluidic liquid metal heater circuit on a thermoresponsive liquid crystal elastomer. The Joule heat of the liquid metal circuit with gravity‐regulated resistance can be programmed by changing the actuator's pose to induce the flow of liquid metal. The actuator can autonomously adjust its bending degree by the dynamic interaction between its thermomechanical response and gravity. A gravity‐interactive soft gripper is also created with controllable grasping and releasing by rotating the actuator. Moreover, it is demonstrated that self‐regulated oscillation motion can be achieved by interfacing the actuator with a monostable tape spring, allowing the electronics‐free control of a bionic walker. This work paves the avenue for the development of liquid metal‐based reconfigurable electronics and electronics‐free soft robots that can perceive gravity or acceleration.

## Graphical abstract

Gravity‐responsive soft actuators are created using liquid metal and thermoresponsive liquid crystal elastomer. The Joule heat of the liquid metal circuit with gravity‐regulated resistance can be programmed by changing the actuator's pose to induce the flow of liquid metal. A gravity‐adaptive actuator, a gravity‐interactive gripper, and a self‐regulated snapping oscillator or walker are also demonstrated.

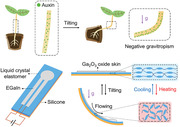

## 1 Introduction

Self‐regulation or homeostasis is ubiquitous and indispensable for all living organisms to adapt to environmental change for survival. For example, plants embody fantasizing phototropism and gravitropism to adjust growth direction to maximize their energy capture.^[^ ^1^ ^]^ Carnivorous plants such as Venus Flytrap can rapidly close their leaves in response to mechanical stimulation to capture prey.^[^ ^2^, ^3^ ^]^ These intelligent plant motions are a result of complex self‐regulating biological feedback to external stimuli, despite lacking nerves and muscles.^[^ ^4^ ^]^ Lessons and inspiration from plant growth have motivated researchers to create self‐regulated soft robots capable of autonomously responding to external stimuli.^[^ ^5^, ^6^, ^7^ ^]^ Different from traditional robots relying on computational intelligence, a few plant‐inspired soft robots can complete complex tasks such as on‐demand gripping,^[^ ^8^, ^9^ ^]^ tropic movement,^[^ ^10^ ^]^ self‐sustained oscillation,^[^ ^11^, ^12^ ^]^ and self‐directed energy capture^[^ ^10^, ^13^, ^14^ ^]^ without the use of any hard electronic controllers. These electronics‐free, autonomous soft robots with distributed intelligence facilitate the miniaturization of robot bodies, and improve their environmental adaptability, paving the way to creating intelligent entities beyond nature for bionics and biomedicine.

Plant‐inspired soft robots have been designed to mimic various botanic responsive motions such as phototropism,^[^ ^15^, ^16^, ^17^ ^]^ hygroscopic motion,^[^ ^18^, ^19^, ^20^, ^21^, ^22^ ^]^ and thigmotropism.^[^ ^9^, ^23^ ^]^ However, these motions frequently depend on artificial stimuli with variable parameters in time or intensity, such as directed light,^[^ ^17^ ^]^ humidity gradient,^[^ ^24^ ^]^ and selective heating.^[^ ^25^, ^26^, ^27^ ^]^ Thus, existing plant‐inspired robots have to be operated in a specific interaction environment that limits their adaptability and potential use. Unlike the abovementioned motions, plants have unique gravitropism in response to the invariable stimulus of gravity. This gravitropism inspired us to create intelligent soft robots regulated by the gravitational field to free autonomous robots from structured environments. Moreover, since gravity is a ubiquitous source, the use of the gravitational field to control the robot's shape morphing could simplify the exertion of external stimuli in a cost and energy‐effective way. More importantly, gravity‐responsive soft robots may inspire the design of acceleration‐regulated intelligent systems, holding great promise for applications in prosthetics, space exploration, human‐machine interaction, and so forth.

Gravity‐induced liquid flow is also a ubiquitous phenomenon that inspired us to create soft actuators with gravitropism in liquid metal (LM)‐actuated systems. Herein, we utilized the unique fluidity of gallium‐based LM in its oxide skin to create a flexible electric heater with programmable resistance regulated by gravity. Assisted by our developed magnetic printing method,^[^ ^28^ ^]^ we directly patterned the LM heater circuit on a thermoresponsive liquid crystal elastomer, to create a fully soft and autonomous actuator in a simple yet efficient way. We showed that the dynamic interaction between the thermomechanical response of the actuator and the gravity enabled the actuator with the gravitropism response. Based on this mechanism, we also created a gravity‐adaptive actuator, a gravity‐interactive gripper, and a self‐regulated oscillator and walker, embodying a variety of biological behaviors including homeostasis, response, and oscillation.

## 2 Results and Discussion

### 2.1 Bioinspired Soft Actuators with Gravitropism

**Figure 1:**
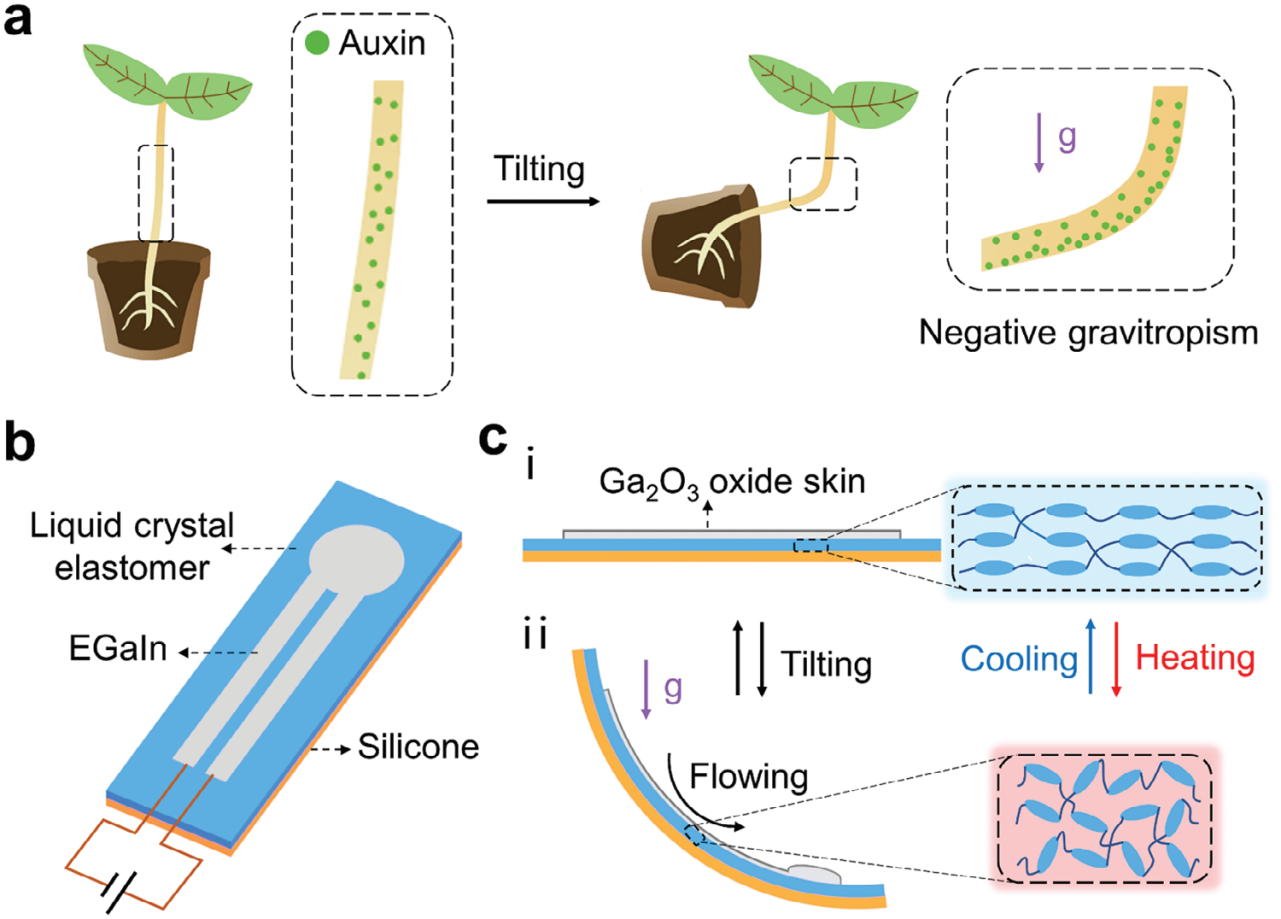
Bioinspired soft actuators with gravitropism. a) Schematic illustration showing the negative gravitropism of plant shoot. b) Schematic illustration showing the bioinspired soft actuator consisting of a liquid crystal elastomer actuator and a gravity‐responsive LM heating circuit. c) Schematic illustration showing the tilt‐induced bending motion of the LM‐LCE actuator under a constant current.

A typical negative gravitropism shows that when the plant is tilted, its shoot bends upwards to keep its growth against the gravity direction (**Figure** **1**a). During this autonomous biological feedback, plants sense the gravity direction by gravity‐sensing cells called statocytes.^[^ ^29^, ^30^ ^]^ These cells respond to gravity by inducing the gradient density of auxin, leading to asymmetrical bending growth.^[^ ^1^, ^31^ ^]^ Herein, statocytes play a key role in both gravity perception and signal transformation, achieving the seamless integration of sensing and actuation in the single plant organ. Given the fascinating LMs having the combination of fluidity and high electric conductivity,^[^ ^32^ ^]^ we envisioned creating an autonomous system based on LMs by utilizing their capabilities in gravity sensing and electrothermal actuation.

To mimic the bending motion of the plant's shoot, we create a thermoresponsive bilayer actuator by coating a passive and adhesive silicone film on the active liquid crystal elastomer (LCE) film (Figure 1b). The LCE can undergo large and reversible deformation upon heating due to the nematic‐isotropic phase transition. The mechanical mismatch between the LCE and the thermostable silicone drives the actuator to bend toward the LCE side when heated (Figure 1c). We then patterned the LM heater circuit on the LCE surface, and the circuit's resistance is dynamic due to the fluidity of LM in its oxide skin. Herein, the LM we used is the eutectic gallium‐indium (EGaIn) with a low melting point of 15.7°C and a high electric conductivity of ≈3.4 × 10^6^ S m^−1^.^[^ ^33^ ^]^ When exposed to air, a self‐limited Ga_2_O_3_ oxide skin (0.5–5 nm in thickness) forms rapidly on the EGaIn surface. The regulation of the oxide skin through chemical or electrochemical reactions has been widely used in creating intelligent LM soft machines.^[^ ^34^, ^35^ ^]^ In this work, this oxide skin can be regarded as a fluid channel wall that allows the LM flow in the patterned LM lines.^[^ ^36^ ^]^ When the actuator is tilted, the LM flows along the circuit under gravity, changing the circuit's cross‐sectional area and resistance (Figure 1c). As a consistent electric current is applied to the circuit, the Joule heat of the circuit and the bending degree of the actuator can be programmed just by tilting the actuator. For instance, when the actuator is in the horizontal state, the LM circuit with low resistance cannot supply sufficient Joule heat to drive the LCE actuator (Figure 1c‐i). However, the tilted actuator exhibits an obvious bending deformation due to the increased resistance and Joule heat (Figure 1c‐ii).

### 2.2 Fabrication and Characterization of the Gravity‐Responsive LM Circuit

Direct printing of LM is challenging due to its unique features such as high surface tension of ≈ 624 mN m^−1^, surface oxidation, and low wettability to substrates.^[^ ^37^, ^38^, ^39^ ^]^ Here, we utilized the magnetic printing method to overcome this challenge and achieved direct printing of LM.^[^ ^28^ ^]^ In a typical operation, we dispersed the magnetic nickel (Ni) microparticle into the LM by mechanical stirring, followed by magnetically spreading LM over the LCE. Under the applied magnetic field, the LM was passively attracted and adhered to the substrate. We then removed Ni microparticles by a magnet and absorbed the excess flowable LM using a syringe, leaving a thin LM film on the LCE surface. The detailed printing procedures were provided in the Experimental Section and Figures S1,S2 (Supporting Information). Note that the printed LM circuit shows enhanced oxidation compared to the pure LM, which helps to maintain the structure integrity of the circuit by avoiding the breakup of the LM line caused by the high surface tension of EGaIn (Figure S3, Supporting Information).

**Figure 2:**
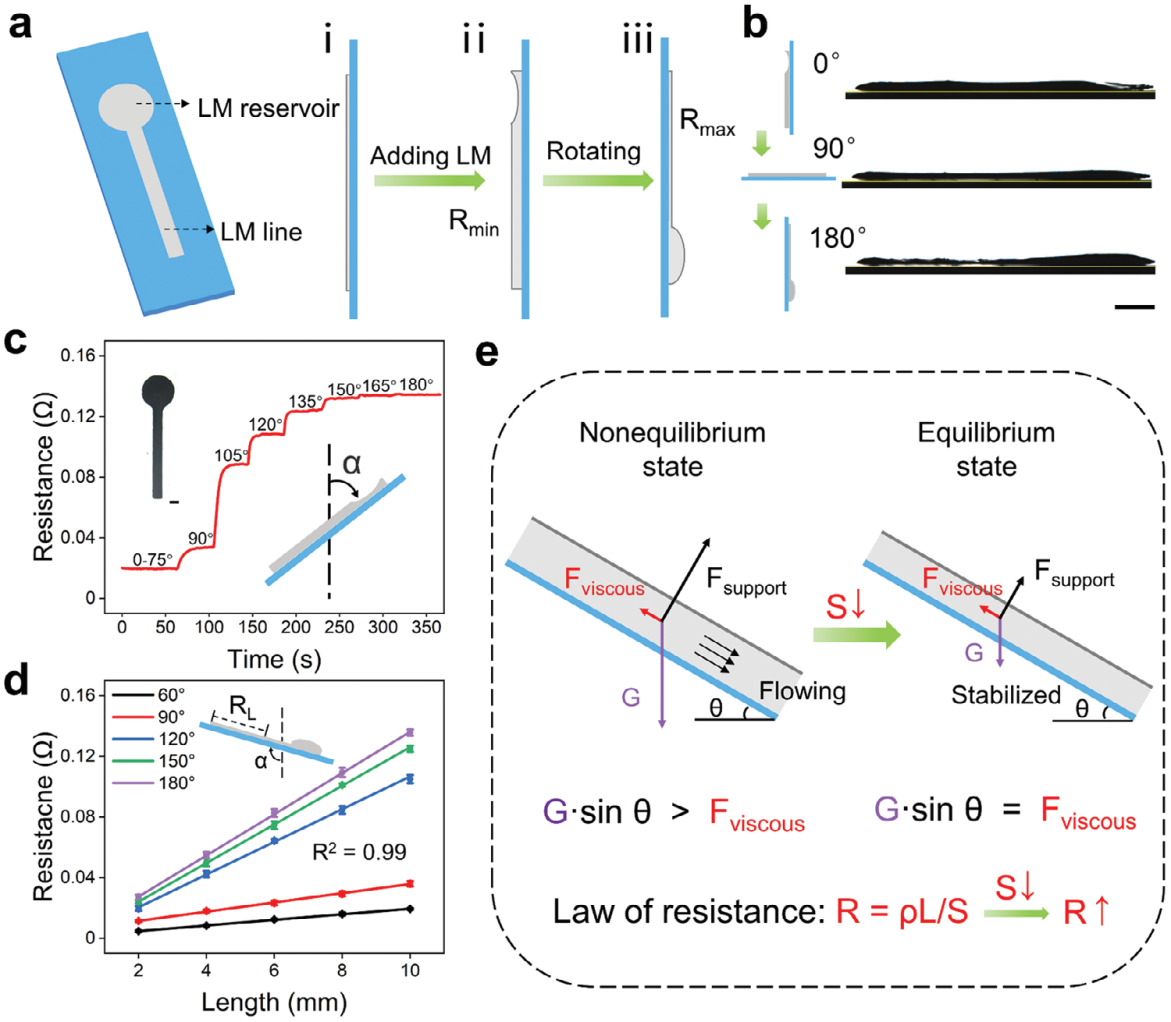
Fabrication and characterization of the gravity‐responsive LM circuit. a) Schematic illustration showing the composition and structure of the gravity‐responsive LM circuit unit. b) Photographs showing the side views of the LM circuit at different tilt angles. c) Resistance of the gravity‐responsive circuit as a function of time. d) Resistance of the gravity‐responsive circuit as a function of length at different tilt angles. e) Free body diagram of the LM in the line. Scale bars: 1 mm.

We designed a typical gravity‐responsive LM circuit unit consisting of a resistive line and a circle reservoir (**Figure** **2**a). To induce moderate flowable LM, we positioned the LM circuit at a tilt angle of 0° where the reservoir was right above the line (Figure 2a‐i) and then added the LM to the circuit until it reached the bottom edge of the circle reservoir (Figure 2a‐ii). In this state, the LM line exhibited the maximum cross‐sectional area but the minimum resistance. After rotating the circuit 180°, the flowable LM was transferred into the reservoir under gravity and the resistance reached maximum (Figure 2a‐iii). We also captured the tilt‐induced liquid flow and the decrease in the thickness of the LM line as the tilt angle increased (Figure 2b; Movie S1, Supporting Information).

We optimized the LM circuit to increase its resistance change by changing the linewidth and the diameter of the reservoir (Figure S4, Supporting Information). For an optimized gravity‐responsive LM circuit, when the tilt angle was less than 90°, the LM accumulated in the line under gravity, leading to the stable R_min_ of 0.02 Ω (Figure 2c; Figure S5, Supporting Information). However, when the tilt angle further increased above 90˚, the LM started to flow from the line to the reservoir, and a synchronous increase in resistance was observed. After each tilt of 15°, the resistance continuously changed and stabilized within tens of seconds, indicating a smooth LM flow in its oxide skin. At the tilt angle of 180°, since the flowable LM transferred from the line into the reservoir, the resistance reached the maximum (R_max_ = 0.134 Ω) which was ≈ 6.7 times the R_min_. It's noted that there was always a layer of LM adhering to the substrate despite the LM flow (Figure S5, Supporting Information), ensuring the connection of the LM circuit even at the tilt angle of 180°. This should be attributed to the magnetic printing that can enhance the adhesion between the LM and substrate.^[^ ^28^ ^]^ In addition, we found that the resistance of the LM line linearly increased with the length at different tilt angles (Figure 2d). This tilt‐dependent fluid flow and uniform resistance/fluid distribution can be explained through the force analysis (Figure 2e). After each tilt, the gravitational force of the LM along the line surpasses the viscous resistance, prompting the LM to flow from the line to the reservoir. With the LM flowing, the thickness of the LM line and the gravitational force gradually decrease until it reaches equilibrium with the viscous resistance again. At the equilibrium state, the tilted LM line of unit length experiences identical influences from gravity, viscous resistance, supporting force, and surface tension. Thus, the LM is evenly distributed in the line, resulting itself a uniform conductor.

It should be noted that the gravity‐induced LM flow in its oxide skin has not been systematically studied. Unlike conventional liquids such as water which usually flows in the fixed channel, the LM flow occurs in its self‐limiting and transformable oxide skin with a thickness of ≈ 6 nm (Figure S6, Supporting Information). To investigate the characters of the LM flow, we approximated the LM line as a uniform cuboid model since the curvature of the LM line was minimal. The relationship between LM resistance and LM thickness can be expressed according to the law of resistance (Figure S7, Supporting Information) and the theoretical resistances coincided with experimental resistances, indicating the reliability of the model. Furthermore, we estimated the gravity‐induced LM flow rate as ≈ 0.026 m s^−1^ based on the rate of resistance change. According to the cuboid model, we calculated the Reynolds number^[^ ^40^ ^]^ of the LM flow as 8.5 (Figure S8, Supporting Information), indicating that the LM flow exhibited laminar characteristics in its oxide skin.

To visually show the gravity‐responsiveness of the LM circuits, we connected the gravity‐responsive circuit in series with a tungsten lamp, in which the lamp luminosity can be adjusted by tilting the circuit (Figure S9; Movie S2, Supporting Information). To investigate the repeatability of the resistance change, we fixed the LM circuit on a continuously rotating platform. It exhibited stable and periodic resistance change during hundreds of cycles (Figure S10, Supporting Information), indicating its good repeatability. We also investigated the electrical stability of the gravity‐responsive LM circuit by applying an electric current of 1.0 A to the circuit for 1 h (Figure S11, Supporting Information). During this process, the resistance of the tilted LM circuit remained invariable, demonstrating its good electrical stability. Moreover, the LM showed a low‐temperature coefficient of resistivity (≈ 10^−3^ per °C)^[^ ^41^ ^]^ and stable viscosity over the temperature ranging from 20 to 80°C (Figure S12, Supporting Information), suggesting its good thermal stability. Due to the stable properties and the uniform resistance distribution, the LM line can produce Joule heat stably and uniformly to drive the thermoresponsive actuators (Figure S13, Supporting Information).

### 2.3 Preparation and Characterization of the LM‐LCE Actuator

**Figure 3:**
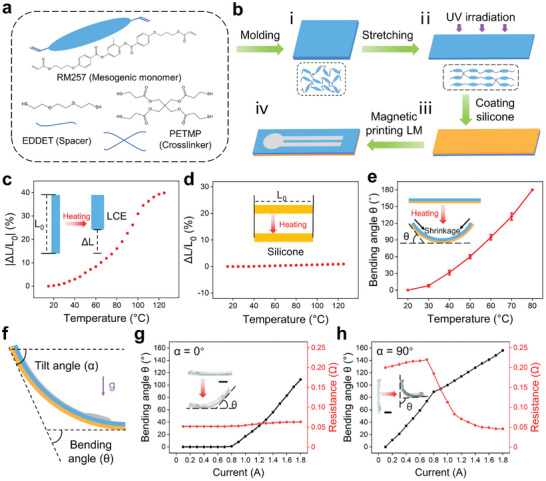
Fabrication and characterization of the gravity‐responsive LM‐LCE actuator. a) Chemical composition of the LCE. b) Fabrication process of the LM‐LCE actuator. ΔL/L_0_ (ΔL = L – L_0_, in which L_0_ is the initial length and L is the length when heated) of c) the LCE layer (25.0 mm × 6.0 mm × 0.3 mm) and d) the silicone layer (25.0 mm × 6.0 mm × 0.4 mm) as a function of temperature. e) Bending angle of the bilayer LCE actuator as a function of temperature. f) Schematic illustration showing the tilt angle (α) and bending angle (θ) of the actuator. Bending angle and resistance of the g) horizontal and h) vertical actuator as a function of current. Scale bars: 5 mm for (g) and (h).

We synthesized the thermoresponsive LCE by a two‐step thiol‐acrylate reaction.^[^ ^42^ ^]^ The chemical components of the LCE consisted of the diacrylate‐based liquid crystal monomer (1,4‐bis‐[4‐(3‐acryloyloxypropyloxy)benzoyloxy]‐2‐methylbenzene, RM257), dithiol flexible spacer (2,2‐(ethylenedioxy)diethanethiol, EDDET), and tetra‐functional thiol crosslinker (pentaerythritol tetrakis(3‐mercapto‐propionate), PETMP), as shown in **Figure** **3**a.^[^ ^43^ ^]^ In addition, dipropylamine (DPA) and 2‐hydroxy‐4′‐(2‐hydroxyethoxy)‐2‐methylpropiophenone (HHMP) were used as the Michael‐addition catalyst and the photoinitiator, respectively. The mixture was infiltrated into the chamber between two glass sides and formed a liquid crystal oligomer after a chain‐extension reaction (Figure 3b‐i). The partially cross‐linked LCE was uniaxially stretched to orient the mesogens into a monodomain state, followed by photopolymerization (Figure 3b‐ii). Subsequently, we coated a layer of adhesive silicone on the LCE surface by blade coating (Figure 3b‐iii). Finally, the gravity‐responsive LM heating circuit was patterned on the other LCE surface by magnetic printing (Figure 3b‐iv; Figure S14, Supporting Information).^[^ ^9^, ^28^, ^44^ ^]^

We first investigated the thermal response of the bilayer actuator itself. The actuation strain of the prepared LCE layer was positively correlated with temperature and reached the maximum value of ≈ 40% at 120°C (Figure 3c). The actuation force per unit length and actuation stress reached 0.19 N mm^−1^ and 0.8 MPa at 100°C, respectively (Figure S15, Supporting Information), indicating the good thermal actuation of the LCE sample. However, the passive layer of silicone demonstrated good thermal stability across a wide temperature range. When the temperature increased from 20 to 120°C, the silicone exhibited a small length change (Figure 3d; Figure S16, Supporting Information) and a low thermal expansion coefficient of ≈ 1.23 × 10^−4^ per °C (Figure S17, Supporting Information). When the bilayer actuator was heated, it exhibited a bending motion due to the mechanical mismatch induced by the difference in thermal expansivity. The bending angle of the bilayer actuator was positively correlated with temperature and reached ≈ 180° at 80°C (Figure 3e). We further disclosed the underlying working mechanism using the relevant systematic theory^[^ ^45^, ^46^ ^]^ and finite element analysis (Figures S18,S19, Supporting Information). The experimental phenomenon coincides well with the theoretical simulation. According to the theory, the bending angle is proportional to the difference in thermal expansion coefficients but inversely proportional to the thickness of the bilayer actuator, which can be used to guide the design of the thermoresponsive bilayer actuator.

Then, we investigated the electrothermal response of the LM‐LCE bilayer actuator. We considered that the Joule heat is determined by the electric current and the resistance, according to Joule's Law (*Q* = *I^2^R* where *Q* is the Joule heat, *I* is the current and *R* is the resistance) and the gravity‐responsive circuit exhibited different resistances at different positions. Thus, we explored the effect of the position on the electrothermal response of the LM‐LCE actuator. Specifically, we applied currents ranging from 0.1 to 1.8 A to the actuator at the horizontal (α = 0°) or vertical (α = 90°) position (Figure 3f). When the actuator was horizontal, the LM circuit exhibited a low initial resistance of 0.073 Ω (Figure 3g). The horizontal actuator started to bend at a current of 0.9 A with a bending angle of 12°. During the bending motion, the LM flowed from the reservoir to the line, resulting in a slight decrease in the resistance from 0.073 to 0.04 Ω. Nevertheless, as the current further increased, the bending angle showed a positive correlation with the current, at a rate of ≈ 110 °per A. Different from the horizontal actuator, the vertical actuator exhibited a high initial resistance of 0.2 Ω (Figure 3h). When the current increased from 0.1 to 0.8 A, the bending angle increased at a ratio of ≈ 128 °per A. The bendin angle reached 90° at the current of 0.8 A. However, when the bending angle further increased, there was a sharp decrease in resistance from 0.2 to 0.046 Ω. As a result, the increase rate of the bending angle decreased to ≈ 67 ° per A. Overall, the horizontal and vertical actuators exhibited distinct electrothermal responses due to the dynamic resistance of the LM circuit, which is affected by both the initial position and bending motion. Moreover, we believe that the previous thermoresponsive bilayer actuators^[^ ^23^, ^47^, ^48^, ^49^ ^]^ may also be endowed with this unique electrothermal response by integrating the gravity‐responsive LM circuit.

### 2.4 Gravity‐Adaptive Actuator with Negative Feedback

**Figure 4:**
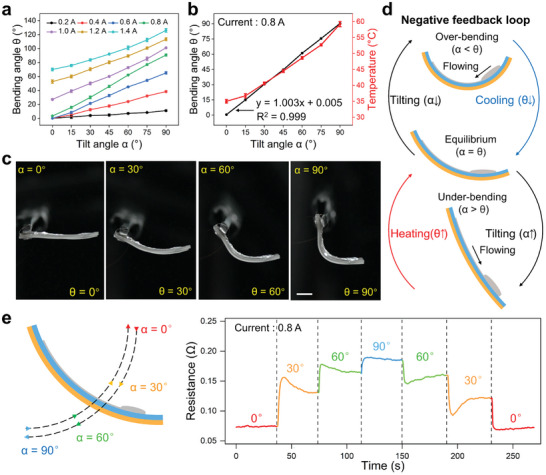
Gravity‐adaptive actuator with negative feedback. a) Bending angle as a function of tilt angle at different currents. b) Bending angle and temperature as a function of tilt angle at the current of 0.8 A. c) Photographs of the tilted actuator at the current of 0.8 A. d) Schematic illustration showing the negative feedback loop of the gravity adaptation. e) Resistance as a function of time at the current of 0.8 A. Scale bar: 5 mm.

In the above experiments, the bending angle was manually controlled by changing the input current. However, plants can autonomously respond to the position change. In particular, they can regulate themselves to the unique equilibrium state, namely, the growth direction is parallel to gravity. We asked whether this LM‐LCE actuator can also reach a certain equilibrium state via self‐regulation. Then, we tilted the actuator at different currents ranging from 0.2 to 1.4 A to investigate their response to tilt, as shown in **Figure** **4**a. Due to the increased Joule heat, the bending angle of the actuator increased with the increasing tilt angle. Surprisingly, we found that the bending angle linearly increased with the tilt angle at 0.8 A with a linear coefficient of 1.003 (Figure 4b). In this case, an interesting phenomenon was observed the bending angle kept consistent with the tilt angle, and the actuator's end stabilized in the horizontal position perpendicular to gravity, as shown in Figure 4c and Movie S3 (Supporting Information).

We believe that there was a self‐established negative feedback loop allowing this gravity‐induced self‐regulatory bending motion. Specifically, when the actuator was at the over‐bending (α < θ) or under‐bending (α > θ) state, the LM flowed to the line or reservoir, thus causing a decrease or increase in the bending angle (Figure 4d). Thus, the actuator can stabilize at the equilibrium state (α = θ). To prove this hypothesis, we tracked the resistance change when changing the tilt angle, as shown in Figure 4e. When the tilt angle increased, a sharp increase in resistance was observed. Then, the bending angle started to increase, leading to the LM flowing back to the line. Finally, the actuator stopped bending and the circuit's resistance stabilized as the bending angle was as large as the tilt angle. If the tilt angle decreased, the corresponding resistance change was opposite to that induced by the tilt angle increase. We investigated the resistance distribution of the LM circuit on the bending actuator (Figure S20, Supporting Information). Since the LM flowed into the lower part of the line and the reservoir under gravity, the LM resistance change mainly concentrated on the tilted part of the line where the cross‐sectional area was smaller. In addition, this gravity adaptation behavior showed good repeatability during 20 tilt cycles (Figure S21, Supporting Information) due to the good reversibility of LCE thermal actuation and LM flow. Unlike previously reported LM‐based robots that typically rely on changing stimuli such as periodically applied voltage,^[^ ^50^ ^]^ and variable magnetic field,^[^ ^51^, ^52^ ^]^ our soft actuator is driven by a constant current to achieve the physical intelligence of self‐regulated gravitropism.

### 2.5 Gravity‐Interactive Gripper

Inspired by mechanoresponsive plants such as the Venus Flytrap or Mimosa,^[^ ^3^, ^53^ ^]^ a series of interactive soft grippers have been developed, showing promising applications in human‐machine interaction, autonomous capture of objects, and visual logic devices.^[^ ^8^, ^9^, ^23^ ^]^ However, existing human‐interactive grippers rely on continuous manual pressure or strain to maintain the actuation.^[^ ^9^, ^23^ ^]^ Considering gravity is a constant force everywhere, we imagined allowing it in the service of human‐machine interaction systems to address this limitation.

**Figure 5:**
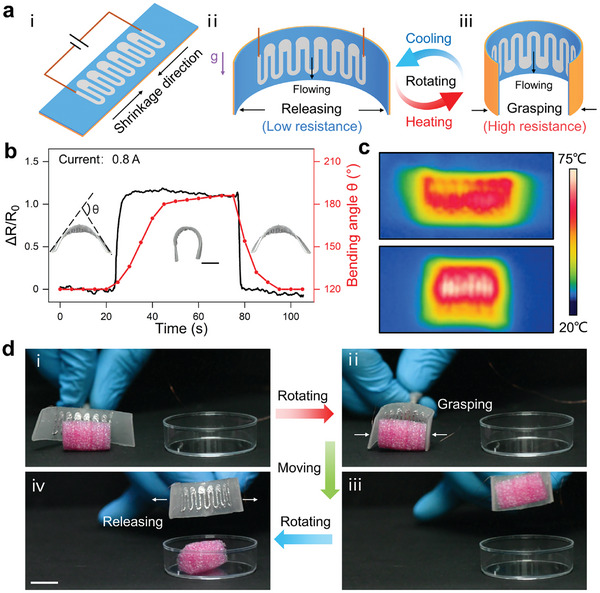
Gravity‐interactive gripper. a) (i‐iii) Schematic illustration showing the gravity‐interactive gripper (i) capable of grasping (ii) and releasing (iii) actuation activated by rotation. b) Synchronous variation in the resistance and bending angle as a function of time at the current of 0.8 A. c) Infrared thermal photographs showing the gripper at opening (up) and closing (bottom) state. d) Photographs showing the gripper manipulating a foam object. Scale bars: 1 cm for (b) and (d).

Herein, we created a novel gravity‐interactive gripper that can autonomously maintain the grasping or releasing actuation after manual rotation, providing a new mode for intelligent human−machine interaction. To create this gripper, we patterned a gravity‐responsive LM circuit composed of twelve heating lines and six reservoirs on a bilayer LCE actuator, as shown in **Figure** **5**a‐i. The resistance of the LM circuit depends on the positional relationship between the LM lines and reservoirs. When the LM lines are over the reservoirs, the LM flows from the lines into the reservoirs under gravity, resulting in the high resistance of the LM circuit (Figure 5a‐ii). On the contrary, the LM circuit exhibits low resistance with the lines under the reservoirs (Figure 5a‐iii). We can manually rotate the gripper to change the positional relationship between the LM lines and reservoirs, thus switching the resistance state of the LM circuit. Under the action of constant current, the low and high resistance induced the grasping (Figure 5a‐ii) and releasing actuation (Figure 5a‐iii), respectively. Notably, considering that the unencapsulated LM circuit was prone to mechanical damage, we designed the extra touching area to grasp the object. This allowed a gap between the object and the LM circuit when grasping the object, preventing the object from touching and damaging the LM circuit (Figure S22, Supporting Information). In a typical gravity‐interactive gripper (Figure S23, Supporting Information), the resistance doubled from 0.45 Ω to 0.98 Ω in ≈ 1 s after rotation of 180°, as shown in Figure 5b. At the current of 0.8 A, the temperature increased from 42°C to 75°C (Figure 5c) and the bending angle increased synchronously from 120° to 180° in ≈ 20 s. Furthermore, we demonstrated object manipulation using this gravity‐interactive gripper (Figure 5d; Movie S4, Supporting Information). At first, the gripper with the reservoirs above the lines maintained the open state (Figure 5d‐i). After rotating 180°, the gripper started to close and autonomously maintained this closing actuation. During this process, the bending angle increased until the gripper clamped the object (Figure 5d‐ii). Then, we transported the object above a container (Figure 5d‐iii) and released the object just by rotating the gripper again (Figure 5d‐iv). In addition, this gravity‐responsive gripper exhibited good grasping‐releasing actuation repeatability during 20 actuation cycles (Figure S24, Supporting Information). Moreover, this soft gripper can also integrate with the traditional rigid robotic arm to endow it with soft grasping ability and achieve autonomous manipulation (Figure S25, Supporting Information).

### 2.6 Self‐Regulated Snapping Oscillator and Walker

Homeostatic oscillations such as neuron impulse, heartbeat, and breathing are widely observed in living organisms. Recently, researchers have made great progress in developing self‐oscillating soft systems, which provide unique opportunities for autonomous robots,^[^ ^16^, ^24^, ^54^ ^]^ transporting devices,^[^ ^12^ ^]^ and smart separation.^[^ ^55^ ^]^ Especially, various electronics‐free robots capable of self‐oscillation have been created by utilizing responsive materials such as the wave‐motion walker,^[^ ^56^ ^]^ self‐sustained swimmer,^[^ ^16^ ^]^ and self‐locomotive hygrobot.^[^ ^57^ ^]^ However, these self‐oscillating robots rely on conditional external stimuli such as directed light^[^ ^14^, ^58^, ^59^ ^]^ and humidity gradient,^[^ ^24^ ^]^ which limit their autonomy and independence. Unlike other changing environmental stimuli, gravity is a constant mechanical stimulus. Thus, the plants and the gravity‐adaptive actuator always regulate themselves to a stabilized equilibrium state. We hypothesize that by disrupting the stability of gravity‐responsive systems, it may be possible to induce oscillation without the need for an external conditioned stimulus.

**Figure 6:**
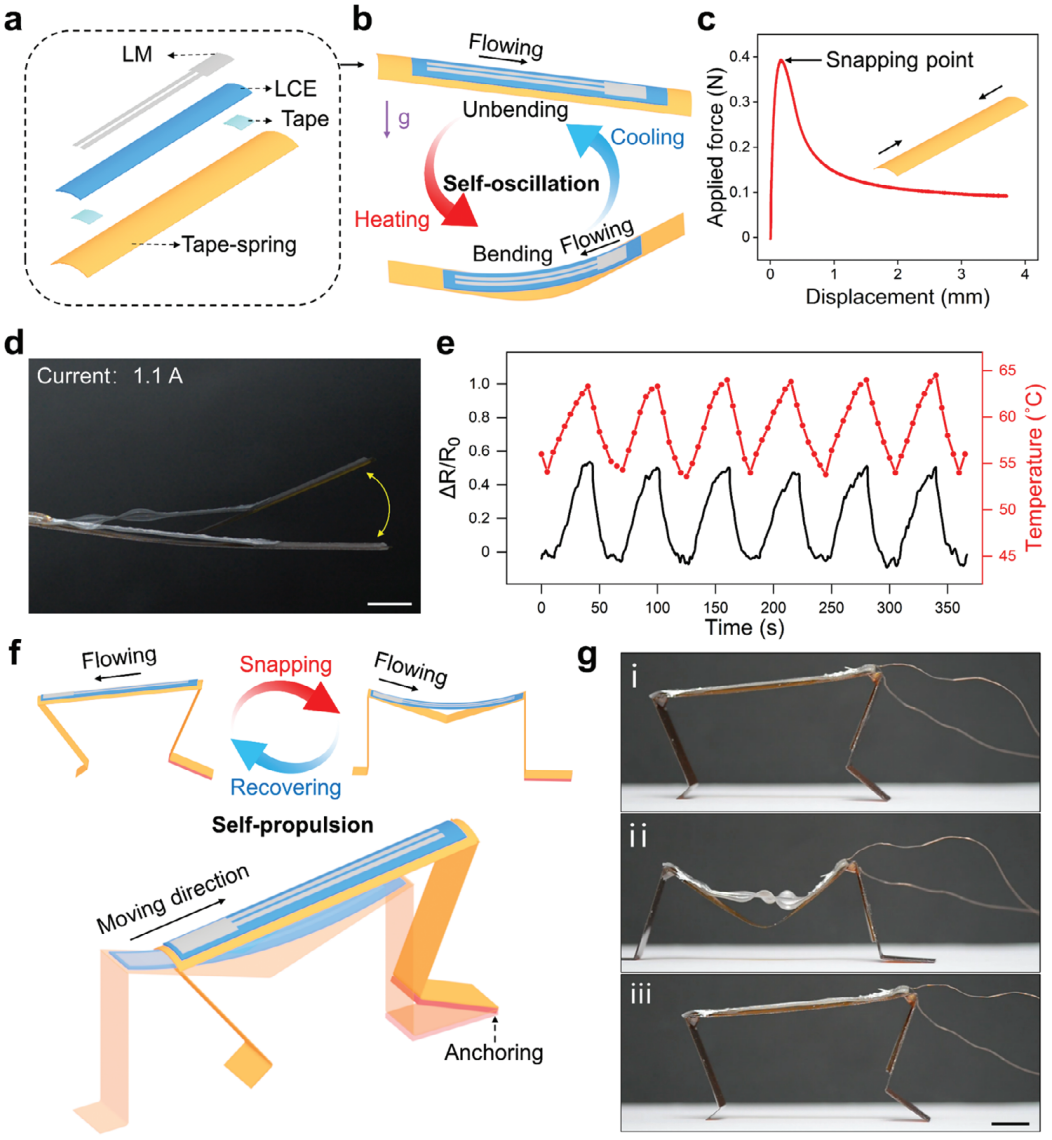
Self‐regulated snapping oscillator and walker. Schematic illustration showing the a) structure and b) working mechanism of the snaping oscillator. c) Force‐displacement curve of the monostable tape spring. d) Superimposed frames of the snaping oscillator (current: 1.1 A). e) Resistance of the LM circuit on the oscillator as a function of time. f) Schematic illustration showing the structure and working mechanism of the snaping walker. g) Photographs of one cycle motion of the snaping walker at the current of 1.1 A. Scale bars: 1 cm for (d) and (g).

Herein, we designed a self‐regulated snaping oscillator by integrating the gravity‐responsive actuator with a monostable polyimide tape spring (**Figure** **6**a,b). The force‐displacement curve of the tape spring indicated its nonlinear mechanic behavior, as shown in Figure 6c. Due to the transition between the positive and negative stiffness, the spring exhibited a snapping motion from an unbending to a bending state when the force was beyond the critical buckling force of ≈ 0.38 N.^[^ ^60^ ^]^ After unloading the external force, the tape spring can spontaneously recover its stable unbending state. This snapping instability and monostability enabled the gravity‐responsive actuator to exhibit oscillation, as shown in Figure 6b. Specifically, the LM on the tilted oscillator flowed from the lines to the reservoir and the resistance increased. Under the action of constant current, the LCE shrank due to the increased Joule heat. Once the shrinkage force of the LCE was beyond the critical buckling force, the tape spring snapped to the bending state. As a result, the LM flowed back to the lines and the temperature decreased. The LCE elongated until the tape spring recovered to the unbending state. Then, the LM flowed to the reservoir again and a new cycle started. A typical snapping oscillator (Figure S26, Supporting Information) showed stable synchronous oscillations in mechanical unbending‐bending motion (Figure 6d; Movie S5, Supporting Information), electric resistance (Figure S27, Supporting Information), and temperature (Figure 6e) under a constant current of 1.1 A.

We further created a self‐regulated walker by installing two asymmetrical claws on the snapping oscillator. The front claw was longer than the back claw to introduce tilt and the front was coated with a layer of silicone to increase its friction (Figure 6f). Due to the tilt of the walker itself, the LM flowed to the reservoir on the horizontal plane and the temperature increased until the snapping motion of the tape spring was triggered (Figure 6g‐i). As the spring rapidly snapped to the bending state with the high‐force output, the front and back claws were launched (Figure 6g‐ii). Then, the LM flowed back to the lines and the LCE elongated. As the spring recovered to the unbending state, the back crawl was pulled forward due to the anchorage of the front crawl. After that, the LM flowed to the reservoir again and the next locomotion cycle started (Figure 6g‐iii). Thus, this snaping walker exhibited self‐propulsion, enabling it to move forward autonomously without requiring external electronic control (Movie S6, Supporting Information). Moreover, this snapping walker showed unique terrain‐sensing ability. To be specific, when the walker was on the tilted plane, the tilt of the plane counteracted the tilt of the walker itself. Thus, the heating lines were lower than the reservoir (Figure S28, Supporting Information), leading to the low resistance of the circuit. The resulting low Joule heat and temperature cannot trigger the snapping motion. The walker remained stationary until the plane returned to the horizontal state (Figure 6g). Note that we can also enable the walker to move on the tilted plane and motionless on the horizontal plane by reversing the positions of the LM lines and the reservoir. With such self‐regulated moving ability, the snapping walker is expected to be applied for environmental exploration. We also imagine endowing it the ability to detect toxic gas pollutants such as hydrogen sulfide by attaching the corresponding test papers to the walker (Figure S29, Supporting Information).

## 3 Conclusion 

In summary, we created soft actuators with gravitropism by patterning the gravity‐responsive LM circuit on the thermoresponsive LCE actuator. Since the LM can flow inside its oxide skin in response to gravity, its resistance and the Joule heat can be reprogrammed by tilting the actuator. Thus, the LM‐LCE actuator exhibited distinct electrothermal motions at different positions. Based on this principle, we demonstrated a gravity‐adaptive actuator capable of autonomously achieving an equilibrium state by establishing a negative feedback loop. In addition, we developed a gravity‐interactive gripper triggered by manual rotation that can maintain the grasping and releasing actuation autonomously. Moreover, we created a self‐regulated snapping oscillator and walker by interfacing a monostable tape spring with the gravity‐responsive actuator. Overall, we rationally leveraged material intelligence to reproduce various self‐regulated biological behaviors in artificial soft actuators without any hard electronic controllers, including gravity adaption, stimuli response, and self‐oscillation. We believe these unique gravity‐responsive soft actuators will pave the avenue for LM‐based reconfigurable electronics and electronics‐free intelligent soft robots.

## 4 Experiment Section

### Preparation of Magnetic LM

The mixture of 74.5 g gallium (99.9%, Dingguan Metal Technology Co., Ltd.) and 25.5 g indium (99.99%, Guifa Alloy Wear‐Resistant Material Co., Ltd.) was heated in an oven at 200°C for 2 h to prepare the EGaIn. The magnetic LM was prepared by mechanically stirring the Ni microparticles (10 wt.%, the average particle size of ≈ 12 µm) into the EGaIn.

### Preparation of Gravity‐Responsive LM Circuit

An adhesive tape, ≈ 50 µm in thickness, was attached to the LCE surface. The tape was patterned using a digital laser engraving machine (CMA‐4030, Han's Yueming) for the *in‐situ* formation of the shadow mask. The laser power was optimized to cut the tape with minimal damage to the substrates. Next, 1 mL magnetic LM was dropped on the mask and the magnetic LM was attracted and dragged by the magnetic field (≈ 300 mT) from a magnet for printing the LM. Excess LM and Ni microparticles were removed using a syringe needle and a magnet, respectively. Finally, the shadow mask was peeled off and added moderate pure LM into the LM circuit.

### Preparation of LCE

The LCE was synthesized via a two‐stage thiol–acrylate Michael addition and photopolymerization reaction (Figure 2a).^[^ ^42^ ^]^ First, the liquid crystal monomer, 1.9 g RM257( Yesheng Chemical Technology Co., Ltd.) and 0.014 g photographinitiatorHMPP(Aladdin) were dissolved into 1.0 mL toluene (Aladdin) at 80°C for 30 min. After cooling down to room temperature, 0.144 g PETMP(Aladdin) and 0.431 g (EDDET (Aladdin) were added into the mixture followed by vortex mixing. 0.284 g DPA(Aladdin, diluted in toluene at a ratio of 1:50) was added to the mixture followed by vortex mixing. The air bubbles in the mixture were removed by degassing in a vacuum chamber. Afterward, the mixture was injected into a rectangular glass cell for a 12 h reaction. Then, the LCE film was taken out from the glass cell and heated at 80°C for 24 h in a vacuum oven to remove the solvent. Finally, the LCE film was uniaxially stretched at 250% strain and exposed to UV light for 1 h.

### Preparation of Gravity‐Adaptive Actuator

Room temperature vulcanization silicone (K‐5905L, Kafuter) was coated on the obtained LCE film surface by blade coating and cured at room temperature for 24 h. Next, the gravity‐responsive LM circuit was patterned on the surface of LCE by magnetic printing.

### Preparation of Oscillator and Walker

The Kapton film with a thickness of 125 µm was rolled up using a glass rod and heated at 80°C for 2 h. Subsequently, the Kapton film was released and cut into the desired shape to fabricate the tape spring. To create the oscillator, the ends of the LM‐LCE actuator were fixed on the Kapton tape spring using adhesive tape. To create the walker, two Kapton claws shaped by cutting and folding were fixed on both ends of the oscillator using adhesive tape. The front claw was blade coated with a layer of adhesive silicone (K‐5905L, Kafuter).

### Characterization

The optical photographs were captured by a digital camera (Nikon D610). The actuation force of LCE was measured using a dynamic mechanical analysis (Q800, TA Instrument). The constant current and voltage were applied by a digital source meter (Keithley 2461). The resistance was measured using the four‐point probe method by the digital multimeter (DMM 6500, Keithley). The temperature and infrared images of the LM circuits were obtained using an infrared camera (FLIR E5). The force‐displacement curve of the tape spring was obtained using a universal mechanical test machine (UTM 2502, SUNS). The thickness of the oxide skin was measured using an atomic force micrograph (AFM, Dimension Icon; Bruker Corporation, USA). The viscosity of LM was measured using a viscometer (LC‐NDJ‐1T, Lichen). The coefficient of thermal expansion was measured using a thermal dilatometer (NETZSCH DIL 402 Expedis).

### Statistical Analysis

Origin 2021 software was used to analyze all of the data statistically. Each set of data was expressed as mean ± standard deviation. The sample size (n) for each statistical analysis was at least three.

## Conflict of Interest

The authors declare no conflict of interest.

## Supporting information

Supporting Information

Supplemental Movie 1

Supplemental Movie 2

Supplemental Movie 3

Supplemental Movie 4

Supplemental Movie 5

Supplemental Movie 6

## Data Availability

The data that support the findings of this study are available from the corresponding author upon reasonable request.
