# Supplementary material for: Soft Robots with Plant‐Inspired Gravitropism Based on Fluidic Liquid Metal
Source: Adv Sci (Weinh). 2024 Mar 6;11(18):2306129. doi: 10.1002/advs.202306129 (PMC11095172; doi:10.1002/advs.202306129)
Supplement: Supplementary file 1 — Supporting Information [file ADVS-11-2306129-s004.pdf]

ADVANCED SCIENCE
Open Access

Supporting Information

for *Adv. Sci.*, DOI 10.1002/advs.202306129

Soft Robots with Plant-Inspired Gravitropism Based on Fluidic Liquid Metal

Gangsheng Chen, Biao Ma, Yi Chen, Yanjie Chen, Jin Zhang and Hong Liu**

Supporting information

Soft Robots with Plant-Inspired Gravitropism Based on Fluidic Liquid Metal

Gangsheng Chen, Biao Ma, Yi Chen, Yanjie Chen, Jin Zhang, and Hong Liu**

State Key Laboratory of Digital Medical Engineering, School of Biological Science and Medical Engineering, Southeast University, Nanjing 210096, China
E-mail: biaom@seu.edu.cn; liuh@seu.edu.cn

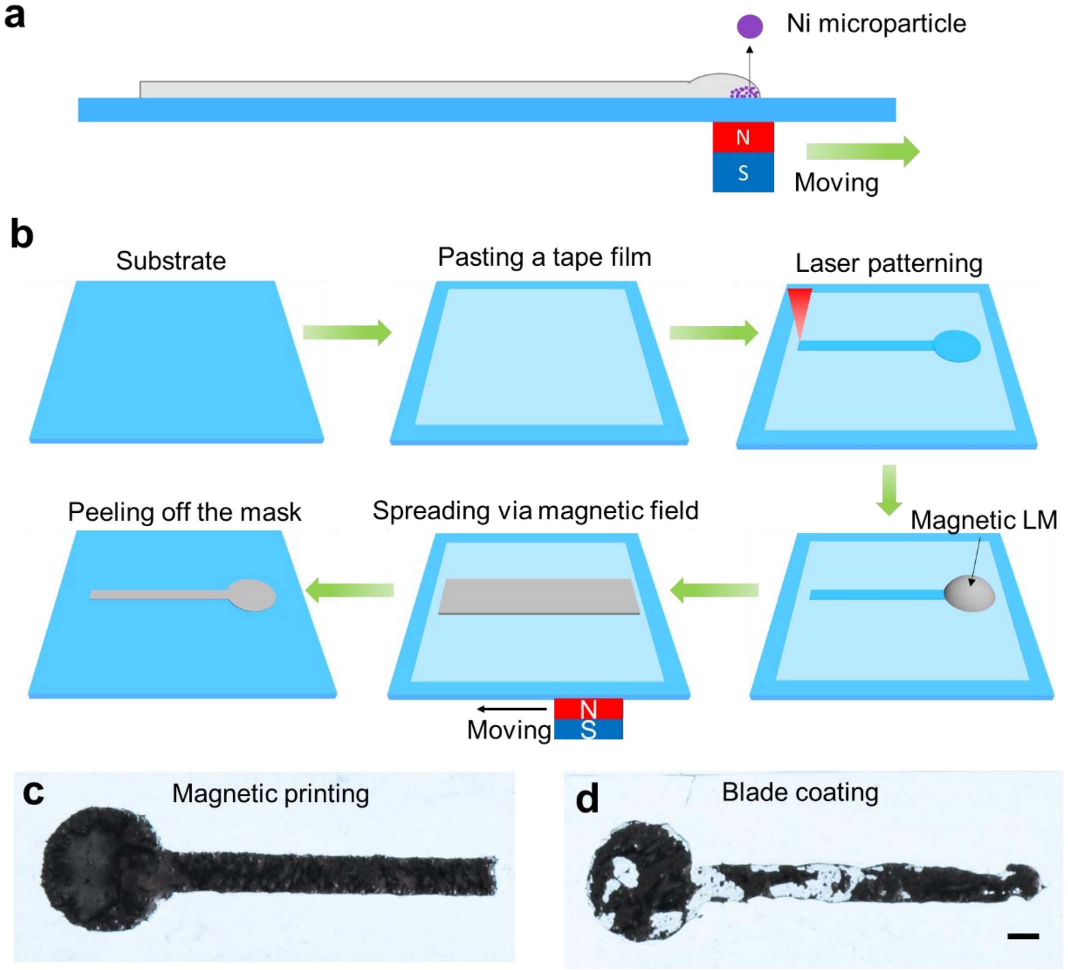

Figure S1. Magnetic printing of liquid metal (LM). (a) Schematic illustration showing manipulation of magnetic LM using a magnet. (b) Schematic illustration showing the LM patterning based on magnetic printing. In a typical operation, a thin adhesive tape was adhered to the substrate, and the tape was cut into the shadow mask by laser engraving. After removing the unwanted parts of the tape, the LM containing nickel microparticles was dropped on the mask and spread by a magnetic field using a permanent magnet. Then, the nickel microparticles (Figure S2) and excess flowable LM were removed using a magnet and a syringe, respectively. Finally, the LM circuit was obtained by peeling off the mask. Photographs showing the LM pattern based on the (c) magnetic printing and (d) blade coating. Scale bar: 1 mm. Magnetic printing can enable the LM pattern to adhere to the substrate. However, the traditional method of blading coating is hard to pattern LM due to the high surface tension of the LM.

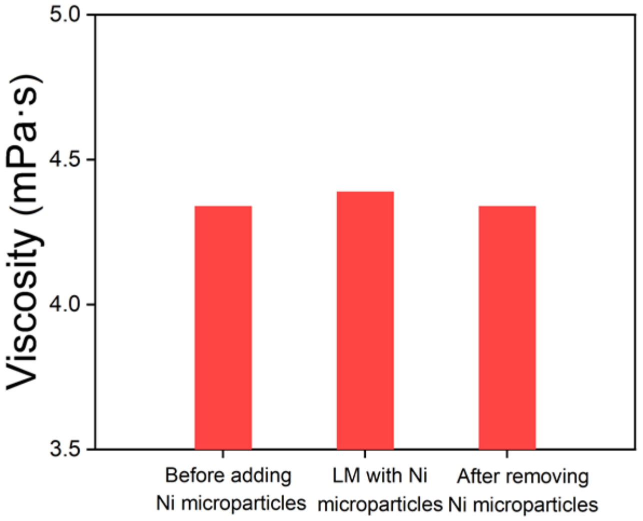

Figure S2. Viscosity of the EGaIn with or without Ni microparticles. The involved Ni microparticles result in a slight increase in the viscosity. After removing the Ni microparticles using a magnet, the viscosity can recover.

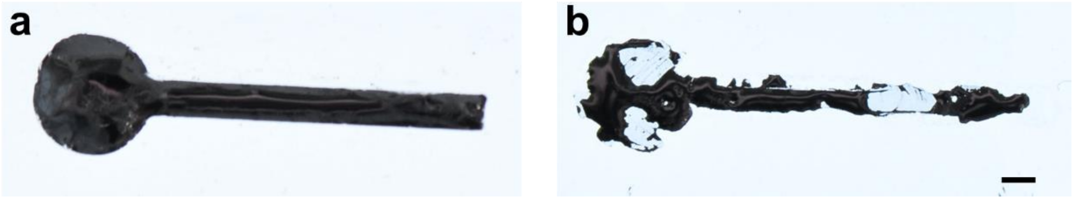

Figure S3. Photograph showing the gravity-responsive LM circuit fabricated using (a) magnetic LM and (b) pure LM. Scale bar: 1 mm. We created the pure LM circuit using a press casting method based on phase transition. To be specific, we press the liquid EGaIn into a mold followed by solidification at -4°C. Then, we took out the solid circuit from the mold and transferred it to a substrate. At room temperature, we found the parent LM was separated from the oxide skin with the EGaIn melting, indicating weak adhesion between them. This also suggests that there should be enhanced adhesion between the oxide skin and parent LM in the stable LM circuit created by magnetic printing.

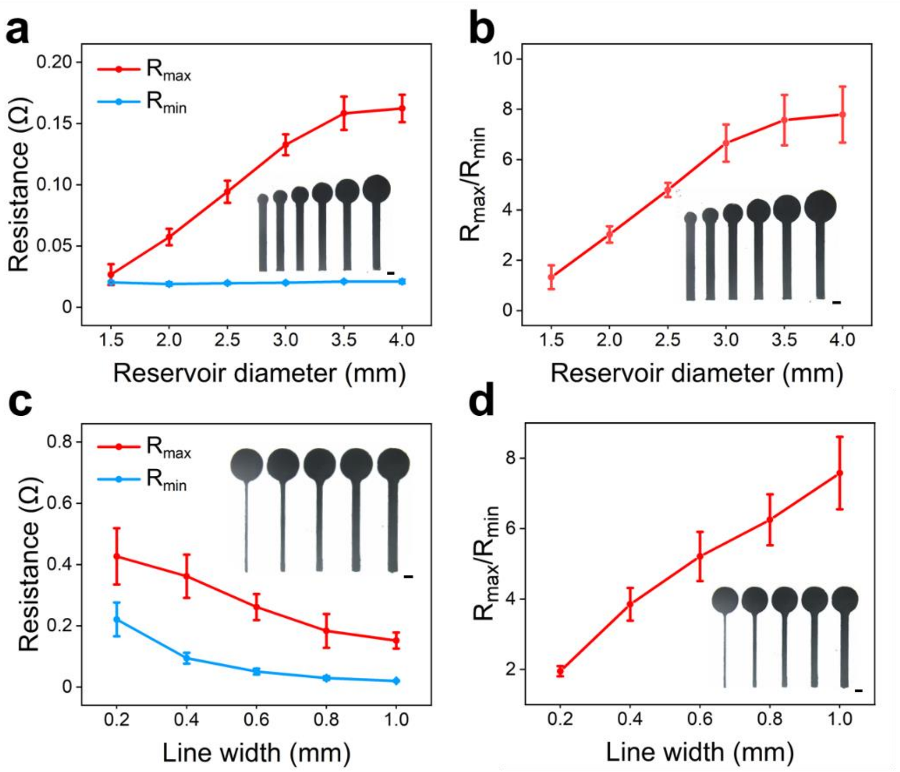

Figure S4. Optimization of parameters for the gravity-responsive circuit. (a) R_{max} and R_{min} and (b) R_{max}/R_{min} of the circuits with fixed line width (W =1.0 mm) and length (L = 10 mm) and varied reservoirs diameter (D = 1.5 mm, 2.0 mm, 2.5 mm, 3.0 mm, 3.5 mm, 4.0 mm). (c) R_{max} and R_{min} and (d) R_{max}/R_{min} of the circuits with fixed reservoir diameter (D = 3.5 mm) and varied line width (L = 10 mm, and W = 0.2 mm, 0.4 mm, 0.6 mm, 0.8 mm, 1.0 mm). Scale bars: 1 mm. The LM line with a width of 1.0 mm and length of 10 mm exhibited an R_{min} of ≈ 0.02 Ω. As the reservoir diameter increased from 1.5 mm to 4.0 mm, the R_{max} increased and peaked at ≈ 0.158 Ω at the diameter of 3.5 mm (Figure S4a and Figure S4b). This is because the larger reservoir can accommodate more LM that flowed from the line. In addition, when the reservoir was large enough, the R_{max}/R_{min} increased with increasing the linewidth, as shown in Figure S4c and Figure S4d. The above experiments suggest a generic principle to create gravity-responsive LM circuits by introducing maximum LM into the lines and ensuring the reservoir large enough to collect the flowable LM. On this basis, the R_{max}/R_{min} can be adjusted by changing the linewidth. The fabrication of the gravity-responsive LM circuits all follow this principle. The circuit with a higher R_{max}/R_{min} possessed a higher sensitivity, exhibiting a greater resistance change at the same tilt angle. The optimized gravity-responsive LN circuit unit (L = 10 mm, W = 1.0 mm, and D =3.5 mm) was for the subsequent tilt test (Figure 2c and d).

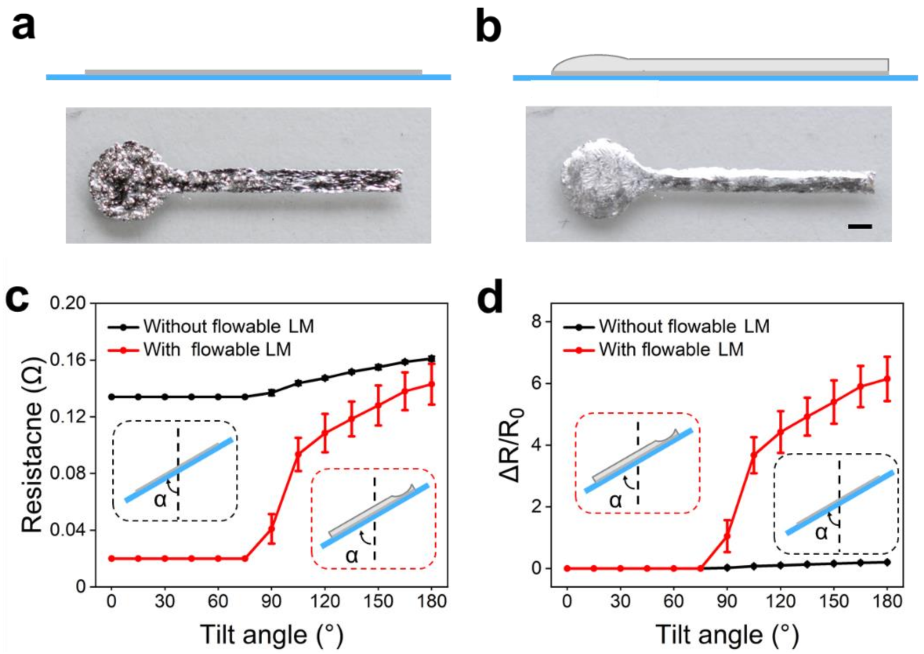

Figure S5. Photographs showing the gravity-responsive LM circuit unit (a) without flowable LM and (b) with flowable LM. Resistance (c) and relative resistance (d) of the LM circuit (L = 10 mm, W = 1.0 mm, and D = 3.5 mm) without and with flowable LM as a function of tilt angle. Scale bar: 1 mm. For the circuit without flowable LM, since the flowable LM cannot be completely removed, the circuit still exhibited slight tilt-induced resistance change. The resistance change (R_{max}/R_{min} = 6.7) of the circuit with flowable LM was increased obviously, compared with that (R_{max}/R_{min} = 1.2) of the circuit without flowable LM. It's noted that despite removing the flowable LM, there was still a layer of LM adhering to the substrate (Figure S5a). This layer of LM ensured the connection of the gravity-responsive LM circuit at different tilt angles.

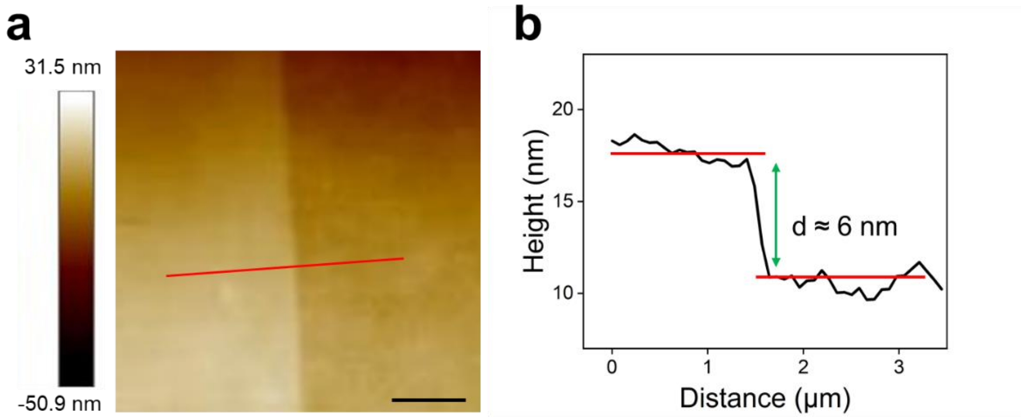

Figure S6. Atomic force micrograph characterization of the Ga_2O_3. (a) AFM topography of the Ga_2O_3 skin. (b) Height profile along the red line. Scale bar: 1 μm.

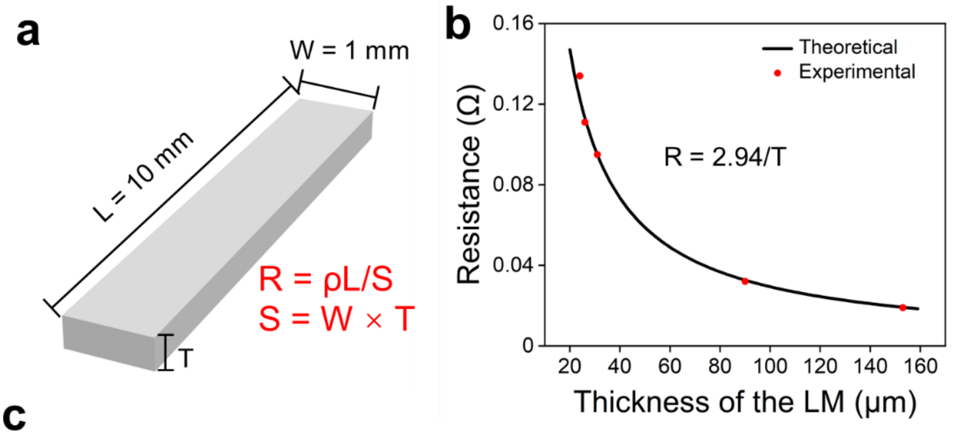

c

Tilt angle (°)	0°	90°	120°	150°	180
Experimental resistance (Ω)	0.020	0.033	0.107	0.125	0.134
Mass of the LM in the line (mg)	9.5	5.6	1.9	1.6	1.5
Thickness of the LM in the line (μm)	153	90	31	26	24
Theoretical resistance (Ω)	0.019	0.032	0.095	0.111	0.122

Figure S7. Simplified calculation model for the LM Line. (a) Schematic illustration showing the uniform cuboid conductor. (b) Theoretical and experimental resistance as a function of the thickness. (c) Table showing the relevant parameters of the LM line at different tilt angles. Ideally, the LM is believed to be evenly distributed in the line of the circuit. We used the swab socked with the ethyl alcohol to wipe the LM line at different tilt angles. By weighing the circuit before and after wiping, the mass of the LM line can be obtained and thus the volume. We can calculate the thickness by dividing the volume by the base area (10 mm^2). The relevant parameters that we used included the density of the EGaIn (6.2×10^3 kg/m^3) and the electrical resistivity of the EGaIn (2.94×10^{-7} Ω·m).

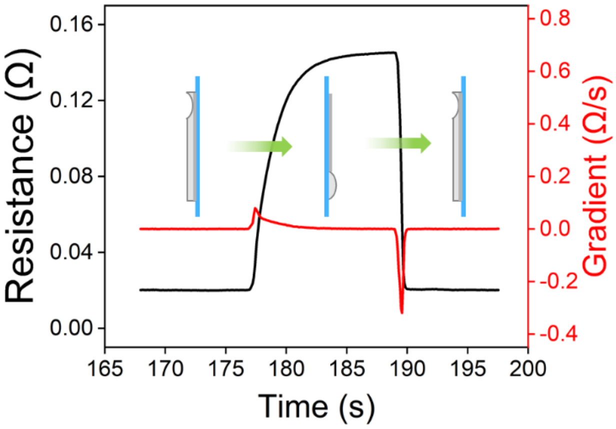

Figure S8. Resistance and resistance change rate as a function of time. The resistance change is 0.125 Ω and the maximum resistance change rate is 0.319 Ω/s. If the resistance changes at the maximum rate, the minimum time for the resistance change is 0.391 s. This means that the flowable LM can fill the line (10 mm in length) at the maximum rate of 0.0256 m/s. The density of the EGaIn ρ is 6.2×10^3 kg/m3 and the viscosity of the EGaIn is 4.3×10^{-3} Pa·s. Based on the cuboid model, the parent flows in a rectangular channel and its characteristic length L is 229 μm. According to

these parameters, we calculated the Reynolds number ($Re = \rho v L/\mu$) as 8.453.

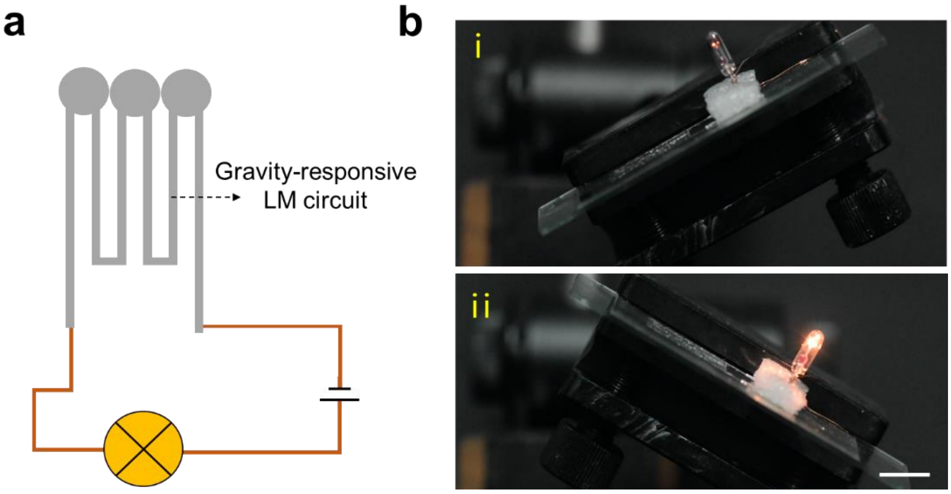

Figure S9. Gravity-responsive LM circuit for adjusting the luminosity of a tungsten lamp. (a) Schematic illustration of the gravity-responsive LM circuit (D = 3.0 mm, W = 0.4 mm, and L = 20 mm, 15 mm, 1.8 mm) connected in series with a tungsten lamp under a constant voltage of 1.5 V. (b) Optical images of the tungsten lamp circuit at different tilt states. During this process, the resistance of the LM circuit changed from 3.1 Ω to 0.9 Ω and the current changed from 0.27 A to 0.3 A. Scale bar: 1 cm.

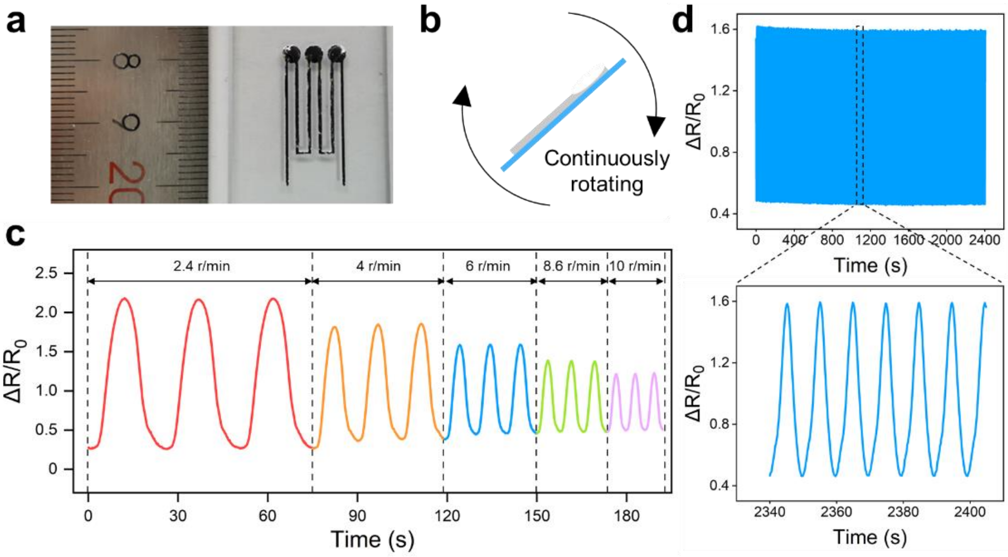

Figure S10. Reversibility of resistance. (a) Optical image of the gravity-responsive LM circuit (D = 3.0 mm, W = 0.4 mm, and L = 20 mm, 15 mm, 1.8 mm). (b) Schematic illustration of the LM circuit on a continuously rotating platform. (c) Relative resistance variation over time in response to the different rotating speeds (2.4 r/min, 4 r/min, 6 r/min, 8.6 r/min, and 10 r/min). As the rotating speed increased, the change period and amplitude of the resistance reduced. (d) Repeatability test of the resistance change.

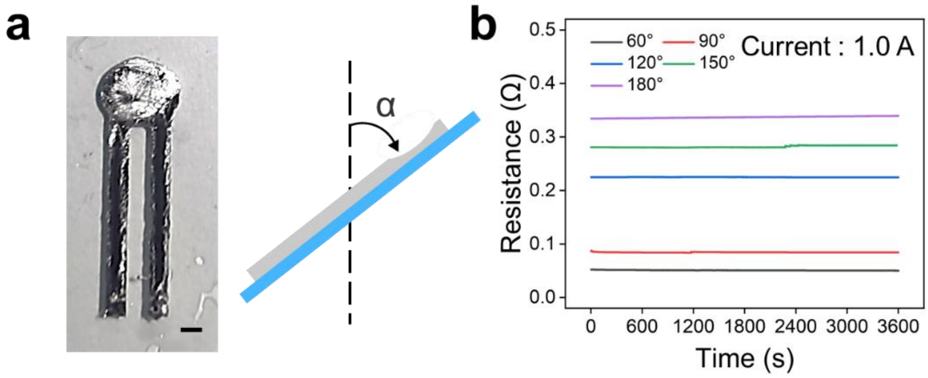

Figure S11. Conductance stability test. (a) Photograph of the LM circuit with two lines (W = 1.0 mm and L =12 mm) and a reservoir (D = 4 mm) and the schematic illustration showing the tilt angle for the test. (b) Resistances of the LM circuit at different tilt angles (1.0 A).

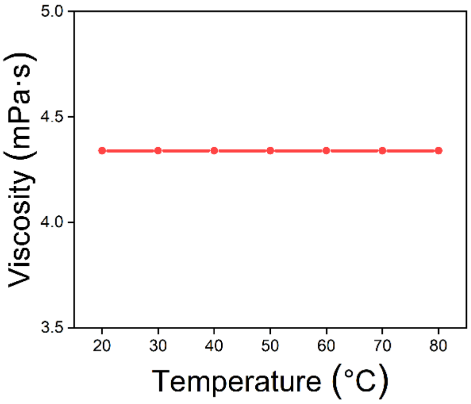

Figure S12. Viscosity of the EGaIn responding to the temperature. The viscosity was 4.34 mPa·s and stable ranging from 20 to 80°C.

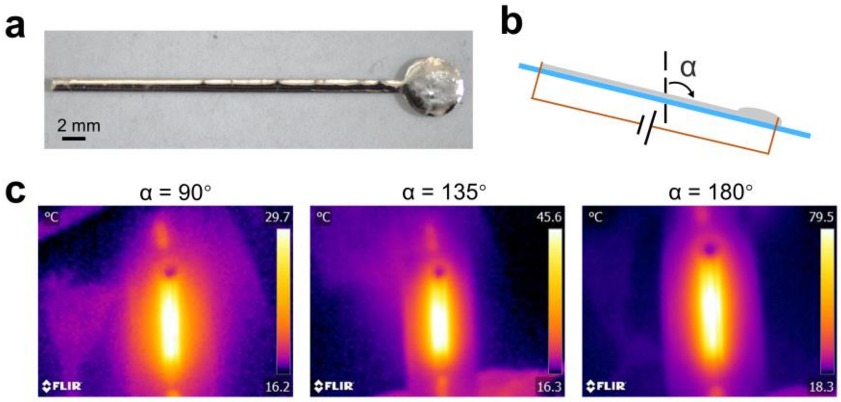

Figure S13. Thermal profile of the LM line. (a) Photograph showing the gravity-responsive circuit composed of a line (W = 1.0 mm and L = 30 mm) and a reservoir (D = 5 mm). (b) Schematic illustration showing the tilt angle (α) of the LM circuit and the external power supply. (c) Infrared thermal images of the LM circuit at different tilt angles when applying the constant current of 2.0 A. The nearly uniform temperature distribution of the LM line indicates uniform heat production.

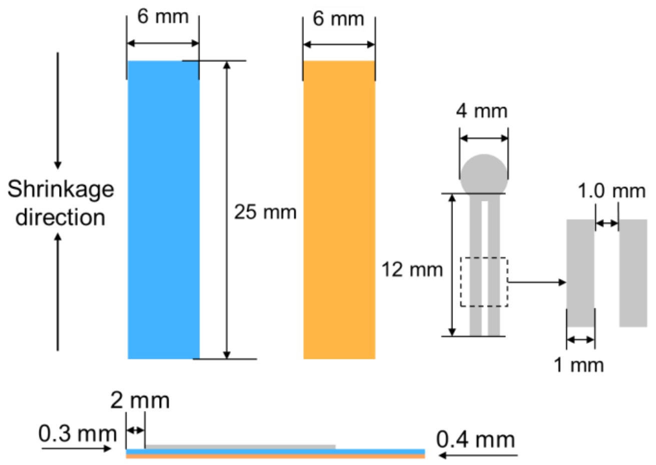

Figure S14. Parameters of components for the gravity-adaptive actuator.

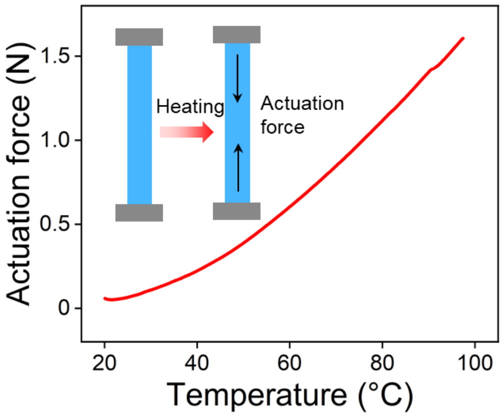

Figure S15. Actuation force of the LCE film (0.4 mm × 5 mm × 8.4 mm) as a function of temperature. The actuation force of the LCE was 1.6 N at 100°C. Thus, the actuation force per unit length and actuation stress were 0.19 N/mm and 0.8 MPa, respectively.

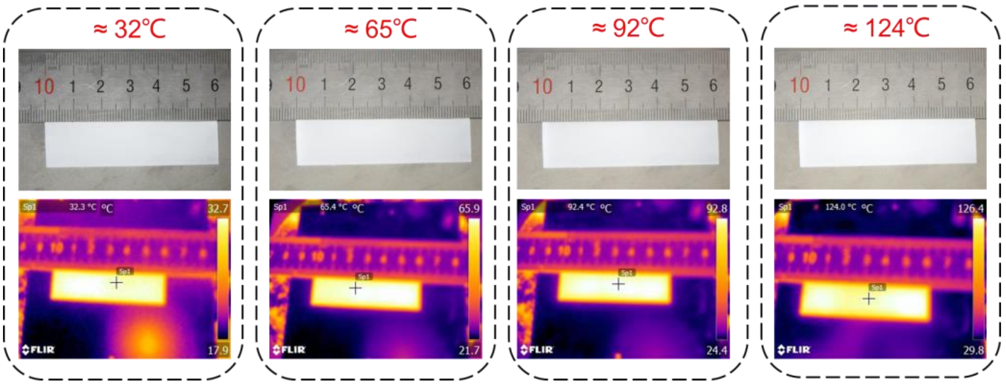

Figure S16. Optical photographs and corresponding infrared thermal photographs of the silicone at different temperatures.

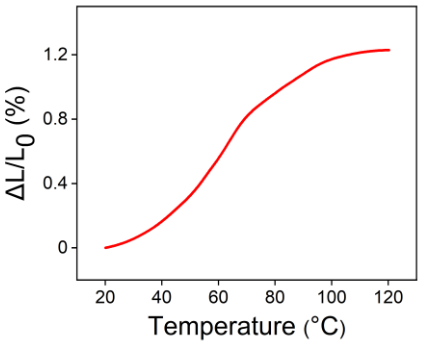

Figure S17. ΔL/L0 of the silicone as a function of temperature. The corresponding coefficient of linear thermal expansion is ≈ 1.23×10^{-4}/℃.

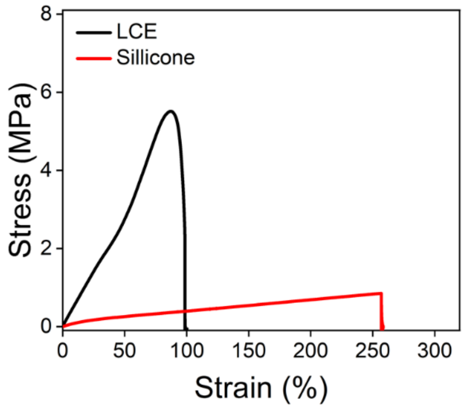

Figure S18. Stress-strain curves of the LCE film and silicone film.

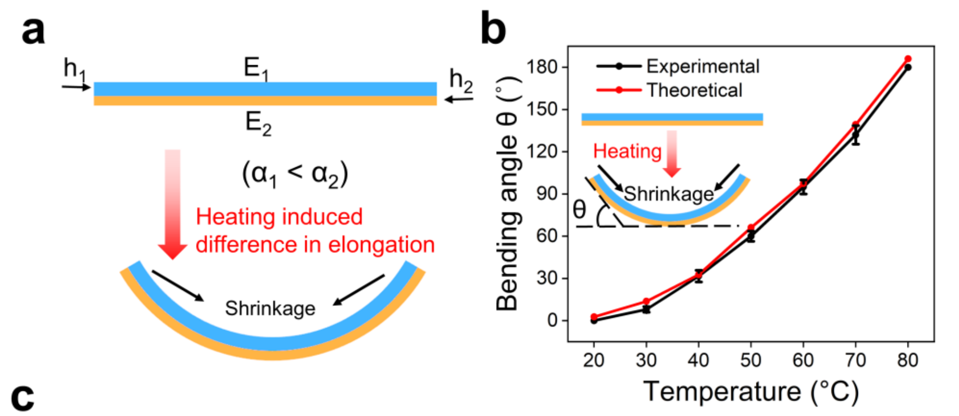

c

Temperature (℃)	20	30	40	50	60	70	80
Experimental bending angle (°)	0	8	32	60	95	132	180
$\alpha_1(T-T_0)$	-0.0024	-0.0119	-0.0298	-0.0571	-0.0833	-0.119	-0.1607
$\alpha_2(T-T_0)$	0	0.0006	0.0017	0.0033	0.0056	0.0082	0.0095
Theoretical bending angle (°) $1095 \times (\alpha_2 - \alpha_1) \times (T-T_0)$	2.6	13.7	32.6	66.1	97.4	139.3	186.4

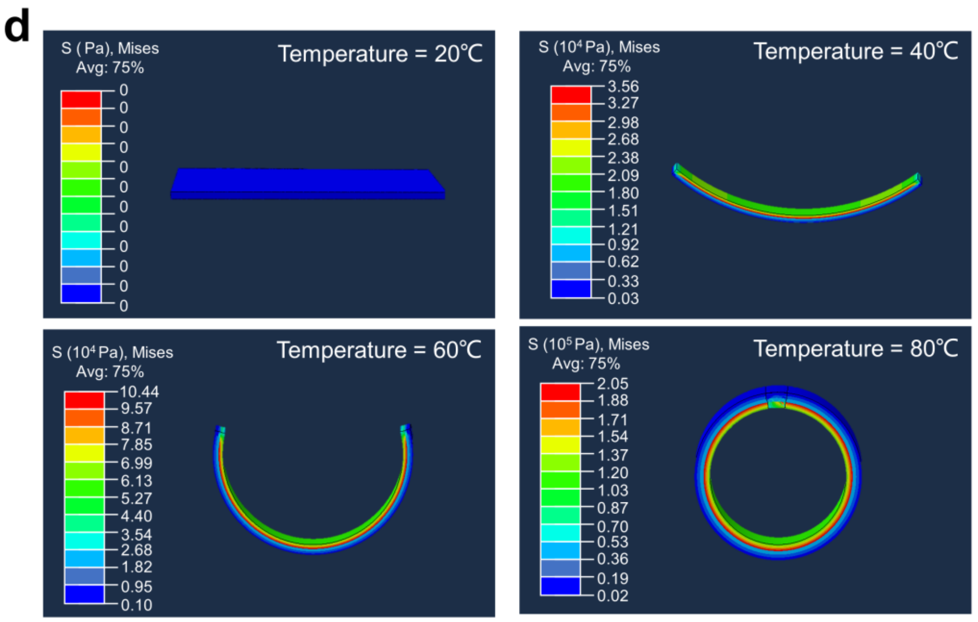

Figure S19. Heating-induced bending motion of the bilayer actuator. (a) Schematic diagram showing the mechanism of the bending motion. (b, c) Experimental and theoretical bending angle as a function of the temperature. (d) ABAQUS simulation showing the bending motion of the bilayer actuator at different temperatures. According to the systematic theory of a cantilever consisting of two layers, which can deal with the uniform bending motion induced by the different thermal expansion coefficients between the two layers, the bending radius ρ of the bilayer actuator can be expressed as

$$\rho = \frac{(h_1+h_2)\left(3(1+m)^2+(1+mn)\left(m^2+\frac{1}{mn}\right)\right)}{6(\alpha_2-\alpha_1)(1+m)^2}\frac{1}{(T-T_0)} \quad m=\frac{h_1}{h_2}, n=\frac{E_1}{E_2}$$

in which h_1 and h_2 are the thicknesses of the two layers, E_1 and E_2 are their elasticity moduli and α_1 and α_2 are their coefficients of expansion, and T_0 is the room temperature. Here, the parameters that we measured and plugged into this formula are the thickness of the LCE layer h_1 = 0.3 mm, the thickness of the silicone layer h_2 = 0.4 mm (Figure S14), the elasticity modulus of the LCE E_1 = 6

× 10^6 Pa, the elasticity modulus of the silicone E_2 = 4 × 10^5 Pa (Figure S17), the room temperature T_0 = 20 °C. In addition, we translated the bending radius ρ to the bending angle using the equation $\theta = L/2\rho \times 57.3°$, where L is the length of the bilayer actuator that is 25 mm. As a result, we obtained the relationship between the temperature T and the bending angle θ, $\theta = 1095 \times (\alpha_2 - \alpha_1) \times (T - T_0)$. $\alpha_1(T - T_0)$ and $\alpha_2(T - T_0)$ can be obtained from Figure 3c and Figure S17. Moreover, the simulation results also accord with the experimental results (Figure S19 b and d).

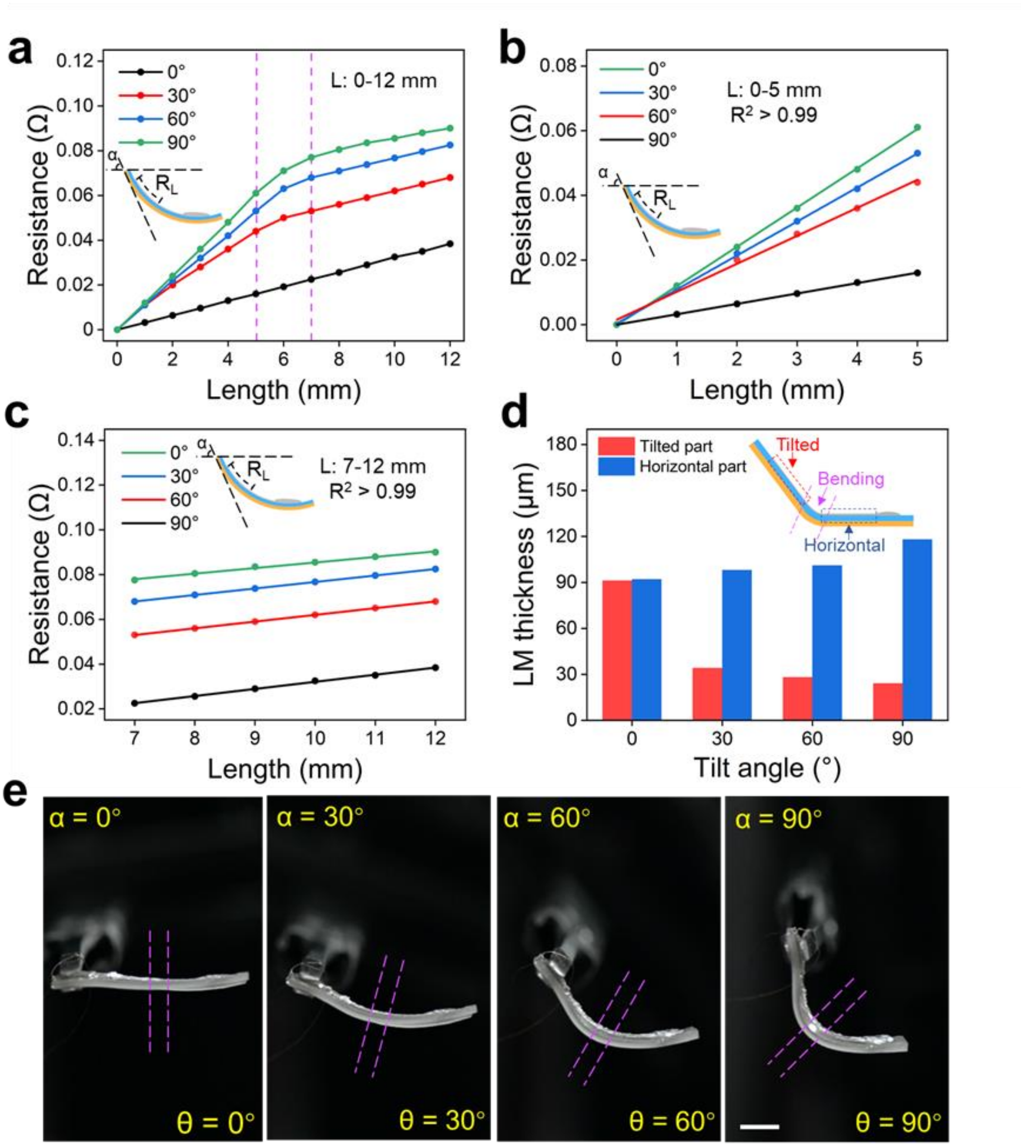

Figure S20. Resistance distribution of the bending LM circuit. Resistance of an LM line (1 mm × 12 mm) on the bending actuator as a function of length ranging (a) from 0 to 12 mm, (b) from 0 to 5 mm, (c) from 7 to 12 mm. (d) Thicknesses of the tilted and horizontal LM line at different tilt angles. Inset: schematic illustration showing the simplified bending motion of the actuator where the heating LM line consists of a tilted, a bending, and a horizontal part. (e) Photographs showing the bending actuator where the parts of the bending line are indicated by the purple dotted line. Scale bar: 5 mm. According to the law of resistance ($R = \rho L/S$), the linear relationship between the resistance and the length indicates that the LM is evenly distributed in the tiled and horizontal part of the line but unevenly distributed in the bending part. The thicknesses of the tilted and horizontal

LM lines are calculated and the former is greater than the latter (Figure S19d). This is because the LM flows from the tilt line to the horizontal line under gravity. Note that the thickness of the LM in the reservoir is the same as that of the horizontal LM line since the reservoir is also horizontal.

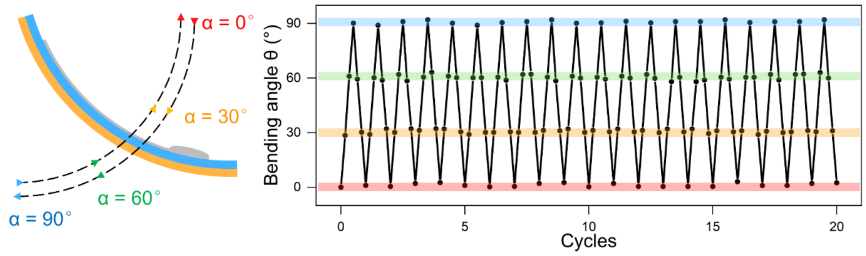

Figure S21. Repeatability test of the gravity-adaptive behavior of the LM-LCE actuator.

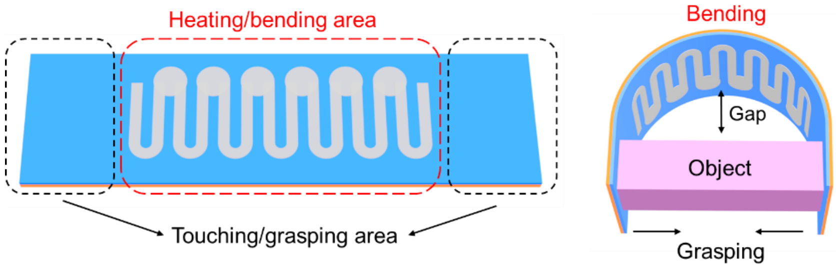

Figure S22. Schematic illustration showing the working mechanism of the gravity-responsive gripper.

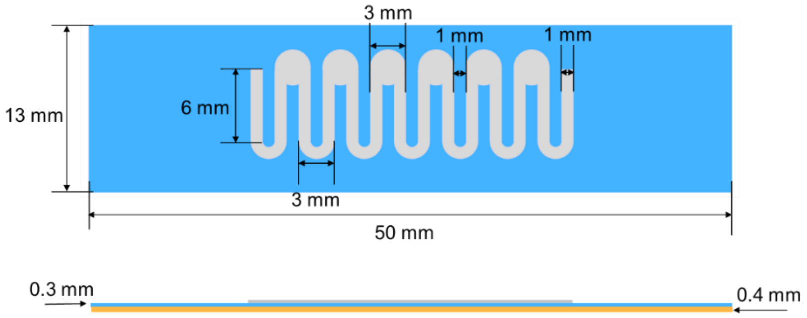

Figure S23. Parameters of components for the gravity-interactive gripper.

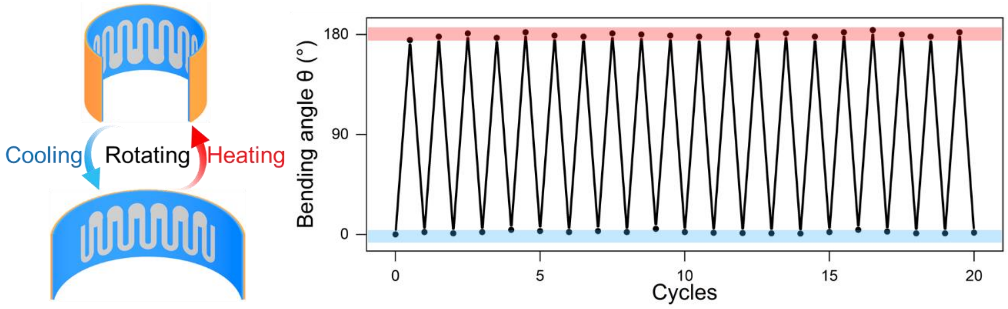

Figure S24. Repeatability test of the releasing-grasping action of the gravity-interactive gripper.

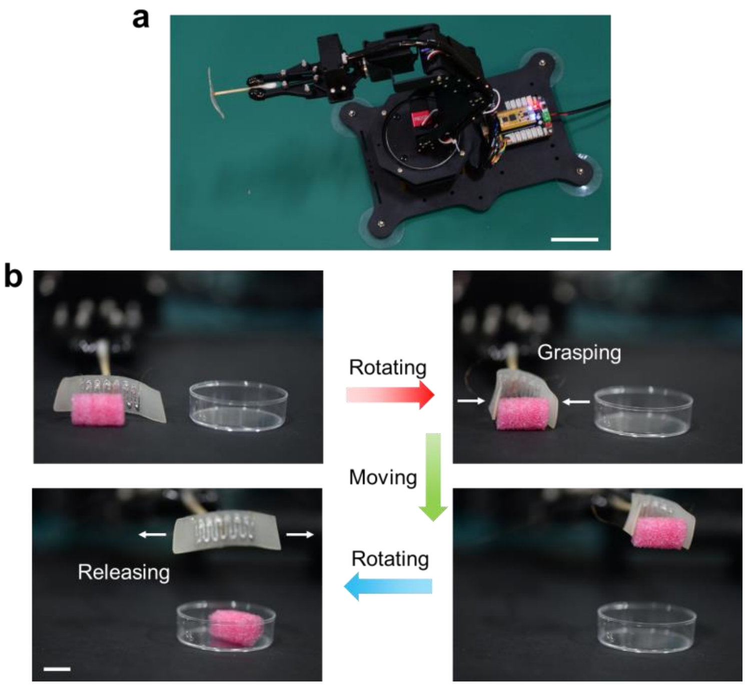

Figure S25. Manipulation of the gravity-interactive gripper using a robotic arm. (a) Photography showing the gripper held by a robotic arm. (b) Photographs of the gripper manipulating a foam object. Scale bars: 5 cm for (a) and 1 cm for (b).

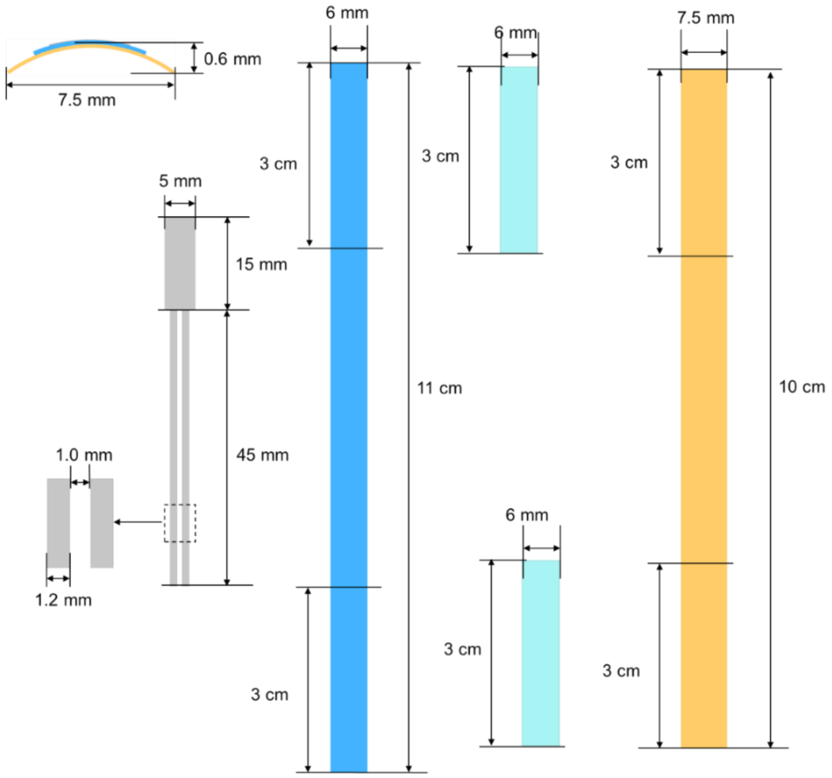

Figure S26. Parameters of components for the LM-LCE oscillator.

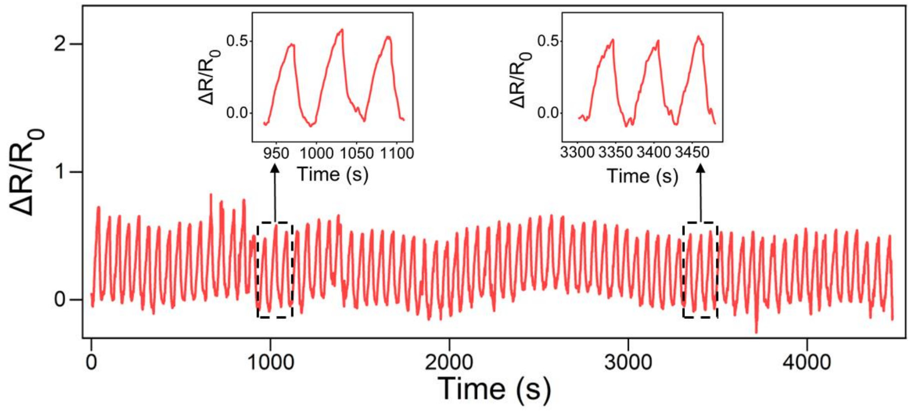

Figure S27. Relative resistance variation of the LM circuit on the oscillator as a function of time.

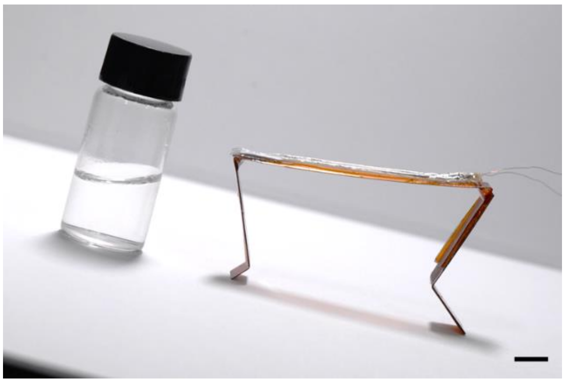

Figure S28. Photograph showing the snapping walker on the tilted plane (current: 1.1 A). Scale bar: 1 cm. On the tilted plane, the heating lines are lower than the reservoir and the flowable LM accumulates in the line, leading to the low resistance. As a result, the snapping motion cannot be triggered by the low heat or temperature and the walker can remain stationary until the plane returns to the horizontal state.

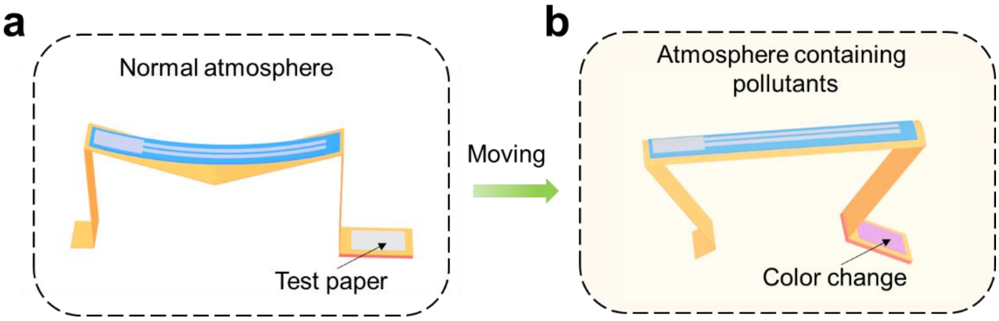

Figure S29. Schematic illustration showing the air pollutant detection of the snapping walker integrated with the test paper. When the walker moves from (a) the normal atmosphere to (b) the atmosphere containing specific pollutants, the color of the test paper will change.
